# Infections in patients taking Rituximab for hematologic malignancies: two-year cohort study

**DOI:** 10.1186/1471-2334-13-317

**Published:** 2013-07-12

**Authors:** Simone Lanini, Aoife C Molloy, Archibald G Prentice, Giuseppe Ippolito, Christopher C Kibbler

**Affiliations:** 1 National Institute for Infectious Diseases, INMI-Lazzaro Spallanzani, Via Portuense, 292, 00149, Rome, Italy; 2 Department of Medical Microbiology, Royal Free Hospital, Pond Street, NW3 2QG, London, UK; 3 Department of Haematology, Royal Free Hospital, Pond Street, NW3 2QG, London, UK

## Abstract

****Background**:**

Rituximab (R) is a chimeric human-murine anti-CD20 monoclonal antibody used to treat B-cell lymphomas. Despite R remarkable activity against malignant cells, there are concerns that R may facilitate the occurrence of infections. This study is aimed to define risk factors for infections, and the potential interaction with time since therapy, in patients undergoing R containing regimens.

****Methods**:**

The study has been designed as a multiple failure events historical cohort including all patients who received a R contain regimen at London Royal Free Hospital between May 2007 and April 2009.

****Result**:**

One-hundred-eighty-one infections occurred among the 113 enrolled patients (overall incidence rate 3.30 per 1000 person-days). Multivariate analysis showed that lymphocyte counts at nadir, graft versus host disease, HIV sero-status and the type of malignancy were all independently associated with the risk of infection. In addition the analysis of the interaction with the time since the start of therapy provided evidence that different risk factors may increase risk of infections in different times.

**Conclusion:**

This study provides preliminary data to describe the association between several patients’ baseline characteristics and infections during therapy with R.

## Background

Rituximab (R) is a chimeric human-murine monoclonal antibody used to treat CD20 positive malignancies and autoimmune diseases. R exerts its activity targeting normal and malignant CD20 positive B cells and allowing a new population of B cells to develop from lymphoid stem cells. It has been shown that R can deplete peripheral B cells while B-cell precursors and mature plasma cells remain unaffected [1]. This may explain the reversibility of R effects on the immune system and its limited effect against multiple myeloma [2].

Given its remarkable activity [3,4], R has been approved since 1998 by European Medicines Agency (EMA) for the treatment of several CD20 positive cancers, including follicular lymphoma, diffuse large B-cell lymphoma and chronic lymphocytic leukaemia [5]. In addition, R is widely used off-label to treat other conditions such as steroid-refractory chronic graft-versus-host disease (GVHD) [6,7].

Although R shows a good safety profile, there are concerns that R may increase the risk of infection in several circumstances [4,8]. Firstly, it has been reported that, patients receiving R as sole drug for maintenance treatment, may experience higher rates of infections than untreated patients [9]. Secondly, the addition of R to multidrug chemotherapy has been associated with a considerable number of severe leucopoenias with unclear clinical significance [4]. Finally the cost effectiveness of R in specific patient groups (e.g. HIV positive) and the actual association of R with re-activation of latent viral infection is yet to be clearly defined [10].

As R affects the immune system in a time-limited and reversible manner, the definition of the timing and the risk factors for infection may help clinicians to improve patients’ outcomes by tailoring interventions to prevent or manage infections based on patients’ individual features.

To estimate the incidence time pattern and potential risk factors for infections associated with R therapy, we collected all positive test results for bacteria, fungi and viruses from patients who started different R regimens in the Haematology department of the Royal Free Hampstead (RFH) NHS Trust and assessed the association of infection with patients’ relevant clinical and epidemiological data.

The report has been written according to the STROBE statement for cohort studies [11].

## Methods

### Setting

The RFH’s haematology department is a 35-bed in-patient unit which care for over 400 patients per year. The unit accepts both patients with malignant and non-malignant conditions and is able to perform allogeneic stem cells transplant.

### Study design

We used a multiple failure events historical cohort study design. Patients were considered at risk of infection from the day of their first R administration until: a) 545 days (18 months) after their enrolment or b) the day they started a new treatment because of failure to respond to therapy or c) the day they died.

### Participants

The list of all patients who underwent R was obtained from the RFH central pharmacy’s database which records patients’ names and the day when each single dose of R was administered. Eligible patients were all adults (aged ≥18) receiving R containing regimens for treatment of hematologic malignancy or for steroid refractory GVHD between May 2007 and April 2009. Patients’ data were obtained from clinical charts and from the Department of Microbiology’s electronic database.

### Outcome and exposure definitions

A clinical case of infection has been defined as: a positive microbiological assay either for bacteria, fungi or viruses which either: a) was associated to newly onset fever >38.3°C; b) led to start a new specific systemic anti-infective therapy; c) led to patient’s immediate hospital admission; d) resulted in the reduction of the intensity of therapeutic protocol for cancer.

For coagulase-negative staphylococci (CNS) and corynebacteria only, two separate positive cultures were required.

A failure event (outcome) was defined as: a) any first clinical case of infection caused by a specific agent in a defined anatomical site of infection (SI); b) the first episode of reactivation of a specific viral latent infection regardless the SI and severity; c) a subsequent clinical case of infection was considered an additional event only if it was caused by a different agent or it was caused by the same agent and occurred 14 days after the earlier episode and the patient had previously tested negative at least once.

Only infections which occurred 2 days or more since the first R administration were included in the analysis.

SIs were defined according to the type of specimen as follows: respiratory tract infection (RTI), blood stream infection (BSI), urinary tract infection (UTI), skin and soft tissues infection (SSTI), gastro-intestinal tract infection (GI) or viral reactivation (specific SI not considered).

Statistical association for outcome and risk factors was assessed for: sex, age (younger than 60 or older than 59), lymphocyte counts at nadir (above or below the population’s lowest quartile), GVHD (yes or no), HIV antibody sero-status (negative or positive), being treated for a relapsing cancer (yes or no), therapy protocol (i.e. R alone or R plus steroids only, R plus chemotherapy, R plus autologous HSCT, R plus allogeneic HSCT), type of disease (i.e.: indolent CD20 lymphoma; aggressive CD20 lymphoma; non-CD20 malignancy), time since first R administration (i.e. first six months, second six months, third six months), mean time in days between subsequent R administrations (as quantitative variable), number of R administrations (as quantitative variable), duration of lymphopenia (cumulative time in days with less than 1×10^9^/L lymphocytes).

### Microbiology

All patients underwent tests on clinical suspicion of infection or when clinically appropriate (according to clinical judgment). In particular, bacteria and fungi were tested by routine cultures methods, respiratory viruses were investigated by multiplex PCR and other viruses were investigated by PCR. Enzyme immunoassay was used for detecting Clostridium diffcile’s toxins A and B. Allogeneic HSCT patients were routinely screened twice weekly for CMV and blood cultures were extended to 10 days after last positive.

### Statistical methods

Crude total rates and those according to aetiology were calculated using a multiple failure model, which considered potential clustering of infections in individual patients, and smoothed hazard functions were plotted.

Association between outcome and potential risk factors was assessed in univariate and multivariate extended Poisson models for recurrent events (gamma frailty model) [12]. The Cox regression model was excluded after a preliminary analysis as hazard proportionality over time could not be assumed.

The best set of variables for the multivariate model was chosen according to simplicity and fitness criteria through a stepwise approach. The degree of complexity was evaluated according to the number of variables and/or inclusion of interaction parameters (i.e.: the fewer the better) while fitness was assessed by likelihood ratio test (LRT) to compare the different models. In this way the more complex model was kept over the simpler one whenever model based LRT p-value was less than 0.100. Given the potential heterogeneity of the study population, interaction between all variables included in the final model was extensively assessed by evaluating departure from multiplicative model. Statistical evidence for association between the outcome and risk factors were inferred either as: no evidence (p ≥ 0.100), weak evidence (0.100 > p ≥ 0.050), good evidence (0.049 > p ≥ 0.010) and very good evidence (p < 0.010).

Analysis of infection incidence time patterns was evaluated by assessing statistical interaction between time as a categorical variable and all other variables included in the final model. In addition, adjusted smoothed hazard functions for all variables included in the final model were plotted.

Clustering of infections at individual patient level (i.e.: within group clustering in the extended Poisson model) was considered statistically significant when the p-value for theta = 0 (i.e. no clustering) was less than 0.05. To calculate a point estimate for the effect of previous infection(s) on the subsequent ones, the number of infection(s) was incorporated, as a quantitative variable, in a standard multivariate Poisson model (i.e. without allowing for clustering) using the same set of variables as for the final model.

STATA 11.2 (StataCorp Texas 77845 USA) package was used for the analysis and to generate plots.

### Ethical statement

Ethical approval for this study was not required under UK research regulations.

## Results

### Participants

Between May 2007 and April 2009 113 patients underwent R regimen at RFH’s haematology unit for a total of 54,923 person days at risk. The description of the cohort, including patients’ epidemiological features, is reported in Table 1.

**Table 1:** Distribution of clinical and epidemiological characteristics of the 113 patients included in the historical cohort and univariate analysis of risk

**Cohort description**						**Univariate risk analysis**	
***Patients’ features***		**Distribution**	**N. of events**	**Person-days at risk**	**Rate (95%CI)**	**RR (95%CI)**	**p-value**
**Sex**	*Male*	65 (57.52%)	121	32675	3.70 (2.71-5.19)	1.00	
	*Female*	48 (42.48%)	60	22248	2.70 (1.71-4.50)	0.73 (0.38-1.40)	0.342
**Age**	<*60*	60 (53.10%)	127	29824	4.26 (3.16-5.86)	**1**.**00**	
	60 or more	53 (47.90%)	54	25099	2.15 (1.33-3.70)	**0.49 (0.26-0.93)**	**0.028**
**Nadir **^**A**^	≥ 0.13 ×10^9^ /L	85 (75.22%)	104	41183	2.53 (1.82-3.59)	**1.00**	
	<0.13 ×10^9^ /L	28 (24.78%)	77	1374	5.60 (3.83-8.51)	**2.04 (1.02-4.05)**	**0.042**
**GVHD**	No	98 (86.73%)	141	47756	2.95 (2.20-4.06)	**1.00**	
	Yes	15 (13.27%)	40	7167	5.58 (3.38-9.79)	**2.18 (0.88-5.44)**	**0.090**
**HIV**	Neg	103 (91.15%)	153	50914	3.01 (2.28-4.05)	**1.00**	
	Pos	10 (8.85%)	28	4009	6.98 (3.45-15.35)	**3.13 (1.06-9.24)**	**0.039**
**Relapse**	No	93 (82.30%)	138	45537	3.03 (2.27-4.13)	1.00	
	Yes	20 (17.70%)	43	9386	4.58 (2.56-8.92)	1.81 (0.79-4.15)	0.163
**Protocol**	R alone	20 (17.70%)	25	10362	2.41 (1.26-5.26)	1.00	
	R and CHT	56 (49.56%)	69	25417	2.71 (1.76-4.37)	1.60 (0.68-3.77)	
	R and auto HSCT	10 (8.85%)	11	545	2.02 (0.69-8.49)	0.84 (0.24-2.93)	
	R and allo HSCT	27 (23.89%)	76	13694	5.55 (3.85-8.27)	2.73 (1.08-6.94)	0.102
**Disease**	Indolent lymph. ^**B**^	43 (38.05%)	45	21934	2.05 (1.28-3.50)	**1.00**	
	Aggressive lymph. ^**C**^	53 (46.90%)	97	25188	3.85 (2.67-5.72)	**2.31 (1.18-4.54)**	
	Non-CD20 malig. ^**D**^	17 (15.05%)	39	7801	5.00 (2.92-9.23)	**2.95 (1.16-7.53)**	**0.024**
**Time**	1^st^ semester	113 ^**E**^	108	18999	5.68 (4.20-7.89)	**1.00**	**<0.001**
	2^nd^ semester	99 ^**E**^	40	18018	2.22 (1.43-3.65)	**0.43 (0.30-0.63)**	
	3^rd^ semester	99 ^**E**^	33	17906	1.84 (1.08-3.41)	**0.36 (0.24-0.53)**	
**Median R doses (iqr)**		4 (2–6)	-	-	-	0.95 (0.84-1.08)	0.471
**Mean days between doses (iqr)**		17 (9–19)	-	-	-	0.99 (0.97-1.02)	0.664
**Median days with <1000 ly (iqr)**		38 (0–220)	-	-	-	1.00 (1.00-1.00)	0.946
**Overall**		113 (100%)	181	54923	3.30 (2.54-4.34)		

***N*** number, ***HSCT*** hematopoietic stem cells transplant, ***HIV*** antibody against human immunodeficiency virus, ***GVHD*** graft versus host disease, ***R*** Rituximab, ***CHT*** chemotherapy, ***IQR*** inter-quartile range, ***RR*** rate ratio, ***95%CI*** 95% confidence interval.

**A**) 0.13x10^9^/L lymphocytes represents the lowest quartiles limit; **B**) This includes: 19 follicular lymphomas; 11 chronic lymphatic leukaemias; 6 lymphoplasmacytic lymphomas; 3 mantle cell lymphomas; 2 multiple myelomas; 2 marginal zone lymphomas. **C**) This includes: 30 diffuse large cell B lymphomas; 8 acute lymphatic leukemias/lymphomas; 7 Burkitt's lymphomas; 4 post transplant lymphomatous diseases; 2 primary central nervous system lymphomas; 2 Castleman's diseases. **D**) This includes: 11 myeloid leukaemias, 5 Hodgkin lymphomas, 1 T-lymphoma; **E**) The figure represent the number of patients at the start of the semester since the first R administration.

### Descriptive analysis

Among the 113 patients enrolled 15 died (i.e. 14 during the first six months and 1 in the last six month period), 39 (34.51%) experienced ≥1 viral infection(s), 49 (43.36%) experience ≥1 bacterial infection(s) and 63 patients (55.75%) experienced one or more infection.

A total of 181 infections were recorded over period for an overall crude incidence rate of 3.30 per 1000 person-days (95%CI 2.54-4.34). Of the 181 infections, 43 occurred while patients were still on R therapy (incidence rate 5.51 per 1000 person-days [95%CI 3.66-8.64] and 138 occurred after the end of therapy with R (incidence rate 2.9 per 1000 person-days [95%CI 2.17-3.96]. The median time to infection since first day of therapy was 18 days [inter-quartile range 7–46]) while the median time to infection since end of therapy was 173 days [inter-quartile range 36–302]. The average number of infections for individual patient was 1.60 (SD 2.24).

BSI were the commonest infections (n = 59; 32.60%) followed by RTI (n = 43; 23.76%), reactivation of latent viral infection (n = 34; 18.78%); UTI (N = 25, 13.81%), SSTI (n = 13; 7.18%) and other infections (this includes: 1 eye infection, 1 middle ear infection, 2 infective diarrhea, 1 acute sinusitis, 2 unknown).

With regard to aetiology, 115 (62.98%) infections were due to bacteria, 62 (34.25%) infections were due to viruses and 5 (2.77%) to other agents (i.e.: 1 aspergillus, 1 mycobacteria and 3 unknown). A complete list of infectious agents is shown in Table 2.

**Table 2:** Aetiology of the 181 cases of infections

**Gram neg. (N = 68)**		**Gram pos. (N = 46)**		**Virus (N = 62)**		**Other (N = 5)**	
E. coli	29	CoNS	26	EBV	17	Aspergillus	1
Pseudomonas	14	S. aureus	9	Rhinovirus	13	Mycobacterium	1
Klebsiella	10	Enterococcus	8	CMV	11	Undefined	3
Serratia	3	Streptococcus	2	Influenza	4		
Moraxella	2	C. difficile	1	PIV	4		
Propionobacterium	2			RSV	3		
Proteus	2			HCV	2		
Stenotrophomonas	2			HSV	2		
Campylobacter	1			Adenovirus	1		
Enterobacter	1			Enterovirus	1		
Fusobacterium	1			HHV6	1		
Haemophilus	1			HHV8	1		
				HMPV	1		
				Norovirus	1		

No infection with mixed aetiology were found.

***E***. ***Coli*** Escherichia coli, ***CoNS*** coagulase negative staphylococci, ***S***. ***aureus*** Staphylococcus aureus, ***C***. ***difficile*** Clostridium difficile, ***EBV*** Epstein-Barr virus, ***CMV*** Cytomegalovirus, ***PIV*** Para-influenza virus, ***RSV*** respiratory syncytial virus, ***HCV*** hepatitis C virus, ***HSV*** Herpes simplex virus 1–2, ***HHV6*** Human herpes virus 6, *HHV8* Human herpes virus 8, ***HMPV*** Human metepneumovirus.

### Crude time analysis

**Figure 1:**
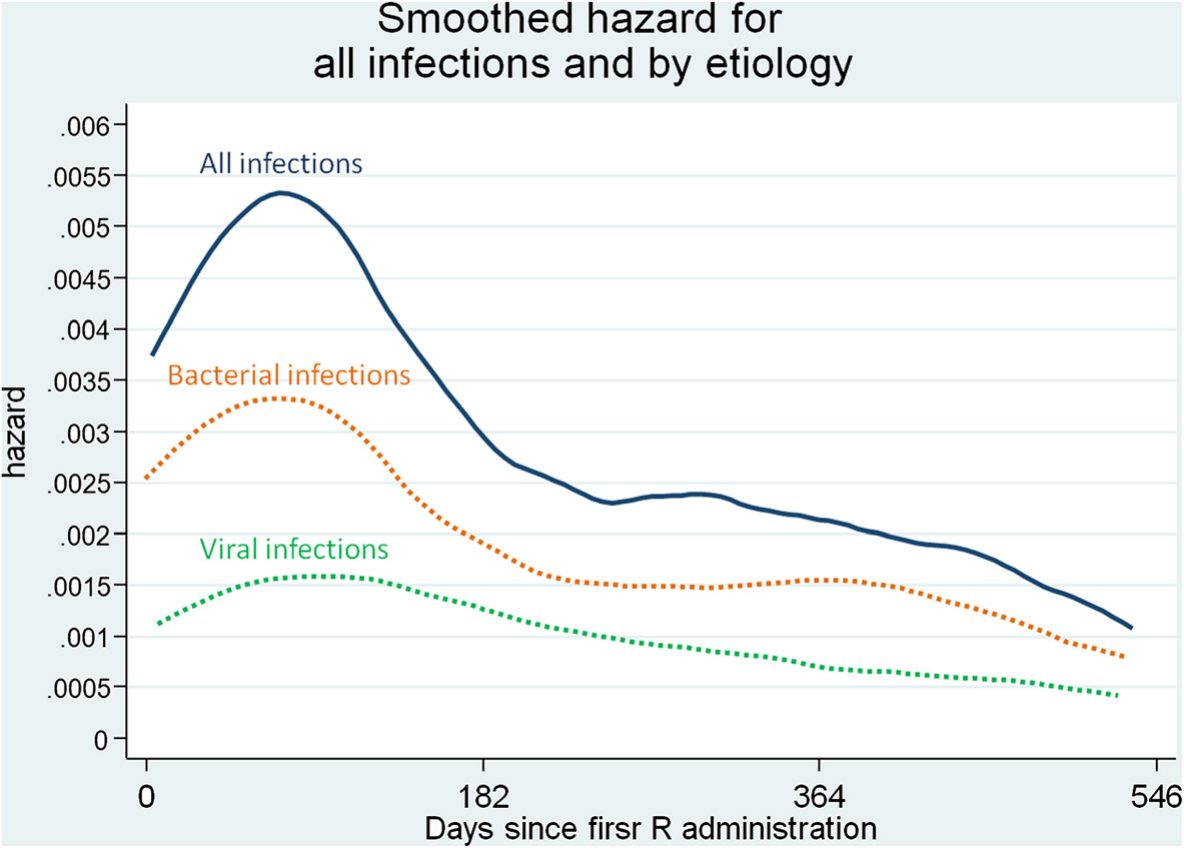
**Smoothed hazard functions which shows time variation of infection incidence from the day of first R administration.** Incidence of infections from all causes is shown by continuous line; incidences of infections by aetiological agent are reported in dotted lines, either viral (green line) or bacterial (orange line).

Crude time analysis provided very good evidence that the global incidence of infections significantly varied over time being 5.68 per 1000 person-days (95%CI 4.20-7.89), 2.22 per 1000 person-days (95%CI 1.43-3.65) and 1.84 per 1000 person-days (95%CI 1.08-3.41) in the first, second and third six month period respectively, after the initiation of R therapy. Figure 1 shows the smoothed hazard for total infections and by aetiology.

### Analysis of risk factors and time adjusted analysis

Table 1 (right side) reports complete results of univariate analysis which shows that weak to very good evidence of association with infections was found for: age (p = 0.028), lymphocyte counts at nadir less than 0.13 × 10^9^/L (i.e. the lowest quartile; p = 0.042), GVHD (p = 0.090), being HIV positive (p = 0.039), suffering from aggressive lymphoma (p = 0.024) and time since first R administration (p < 0.001).

After the stepwise selection process and the assessment of potential interaction, only lymphocyte counts at nadir, GVHD, HIV sero-status and type of malignancy were included as independent variables in the final model (no interaction between the included variables was found).

**Figure 2:**
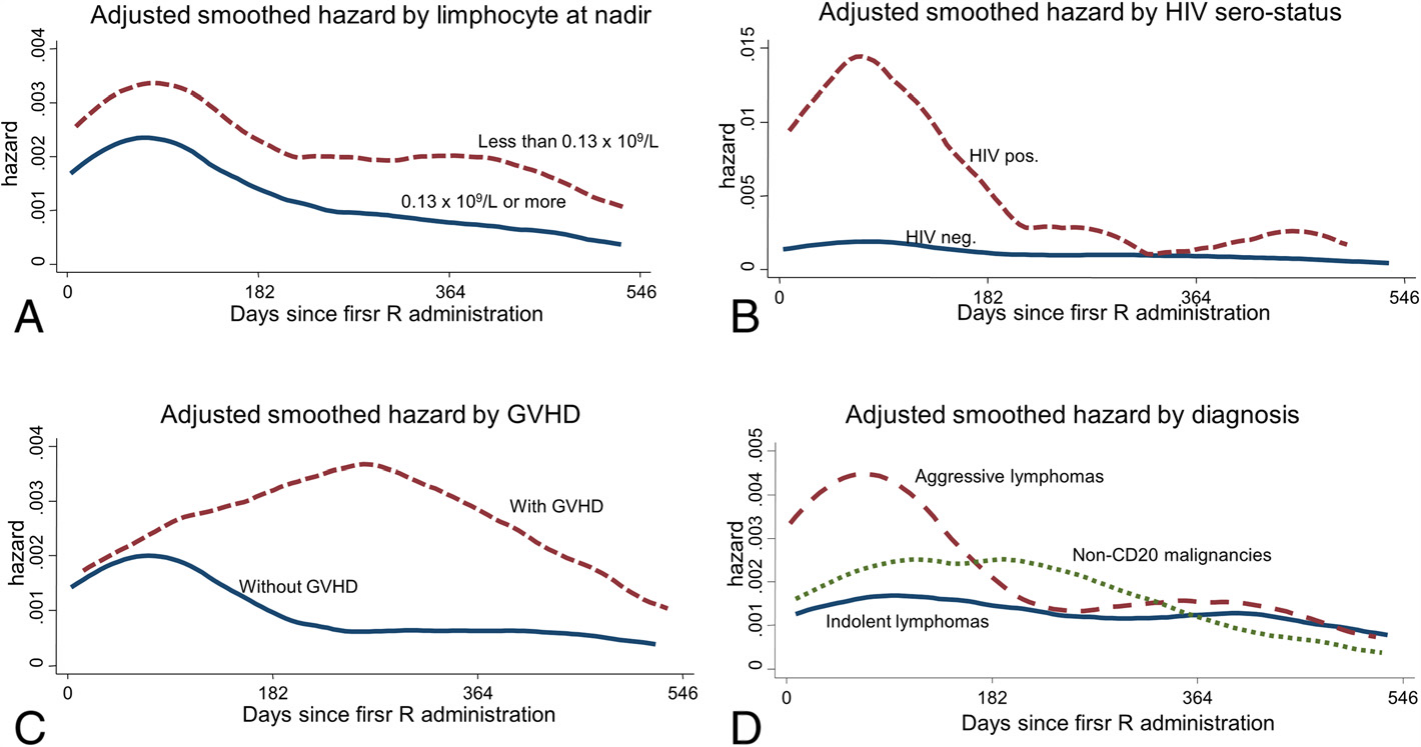
**Adjusted smoothed hazard functions which shows time variation of infection incidence from the day of first R administration according to the 4 different risk factors included in the final model.** This is: lymphocyte counts at nadir **(A)**; HIV sero-status **(B)**; GVHD **(C)**; type of malignancy **(D)**. The hazard function for each specific risk factor is adjusted for the other 3. As reported in the multivariate analysis the graph shows that different risk factors accounted for increased risk of infections in different time periods. In particular, patients in the lowest quartile of lymphocytes counts at nadir were more likely to develop a clinical infection throughout the study period **(A)**; being HIV positive and being affected from aggressive lymphoma seemed to be associated with clinical infections only in the first six months **(B** and **D)** while patients who experienced GVHD were at increased risk of clinical infection from day 183 up to the end of follow-up **(C)**.

The results of multivariate analysis (Table 3) including time as effect modifier (i.e. interaction term) provide evidence that different risk factors accounted for increased risk of infections in different time periods. In particular, patients in the lowest quartile of lymphocyte counts at nadir were more likely to develop an infection throughout the study period (weak to good evidence throughout the 3 time periods). In contrast, being HIV positive and being affected with aggressive lymphoma seemed to be associated with the risk of infections only in the first six months while patients who experienced GVHD were at increased risk of infections from day 183 up to the end of follow-up. Figure 2 shows the adjusted hazard function.

**Table 3:** Multivariate analysis

**Time**	**Risk factor**	**RR**	**p-value**
**First six months period *day 0–182 since first R dose***	Nadir ≥0.13 ×10^9^ /L	1.00	
	Nadir <0.13 ×10^9^ /L	1.89 (1.00-3.60)	**0.051**
	HIV neg.	1.00	
	HIV pos.	**2.92 (1.11-7.65)**	**0.030**
	no GVHD	1.00	
	GVHD	1.49 (0.52-4.27)	0.453
	Indolent lymphoma	1.00	
	Aggressive lymphoma	**2.76 (1.34-5.670**	**0.006**
	Non-CD20 malignancy	1.56 (0.56-4.33)	0.394
**Second six months period *day 183–364 since first R dose***	Nadir ≥0.13 ×10^9^ /L	1.00	
	Nadir <0.13 ×10^9^ /L	**2.68 (1.19-6.04)**	**0.017**
	HIV neg.	1.00	
	HIV pos.	1.21 (0.27-5.38)	0.798
	no GVHD	1.00	
	GVHD	**7.22 (2.38-21.92)**	**<0.001**
	Indolent lymphoma	1.00	
	Aggressive lymphoma	1.21 (0.48-3.02)	0.687
	Non-CD20 malignancy	1.57 (0.49-4.98)	0.447
**Third six months period *day 364–546 since first R dose***	Nadir ≥0.13 ×10^9^ /L	1.00	
	Nadir <0.13 ×10^9^ /L	**2.54 (1.07-6.02)**	**0.034**
	HIV neg.	1.00	
	HIV pos.	1.49 (0.33-6.67)	0.601
	no GVHD	1.00	
	GVHD	**3.65 (1.11-12.04)**	**0.034**
	Indolent lymphoma	1.00	
	Aggressive lymphoma	1.53 (0.60-3.91)	0.371
	Non-CD20 malignancy	0.93 (0.25-3.42)	0.908

The model allows interaction with time since first Rituximab administration and each risk factor. The analysis provides moderate to strong evidence that all the 4 risk factors were associated with the infections in a time dependent manner; i.e.: HIV and type of diagnosis were associated with increased risk of infection in first six months only, GVHD in the second and third time periods while counts of lymphocytes at nadir was associated with increased risk for all the 3 time periods.

***HSCT*** hematopoietic stem cell transplant, ***HIV*** antibody against human immunodeficiency virus, ***GVHD*** graft versus host disease, ***R*** Rituximab, ***CHT*** chemotherapy, ***IQR*** inter-quartile range, ***RR*** rate ratio, ***95%CI*** 95% confidence interval.

### Analysis of infections clustering in individual patients

We found very good evidence of clustering of infections in individual patients (theta = 1.194574; p < 0.001). Point estimates for the effect of previous infection(s) on the subsequent ones, calculated according to standard Poisson regression, were 1.49 (95%CI 1.41-1.57; p < 0.001) and 1.59 (95%CI 1.50-1.68; p < 0.001) in univariate and multivariate model respectively; this is a risk increment of about 60% for the next infection after a previous one.

## Discussion

After more than 10 years of use, R has proven to be a remarkably safe and effective in patients with haematological malignancies [3]. In a previous study [4] we highlighted that the addition of R to standard chemotherapy does not increase the risk of infections, however we found a paucity of data about potential risk factors which may increase the risk of infections in selected groups of patients. The present study was aimed to examine the incidence time pattern and risk factors for infections in patients with different base-line clinical conditions and different hematologic malignancies. Several issues emerge from our analysis.

Firstly we found an infection rate of 3.30 per 1000 person-days with a crude infection risk of 53.75% (63 out 113 experienced ≥ 1 infections) which is higher than the 4.55% to 33.22% reported in the 10 RCTs included in previous meta-analyses [3,4]. This difference may arise form: a) different case mix, as we included frail patients who are more prone to infections than the selected populations included in RCTs (i.e.: 47.89% were affected from aggressive lymphomas, 32.68% received HSCT, 17.67% were relapsing subjects, and 8.50% were HIV positive); b) because clinical trials mainly report prospectively recorded severe infections while we reported clinically relevant infections according to a broader case definition applied on historical data.

However, the overall risk of infection we found is similar to those reported in other historical cohorts including patients with refractory diseases and/or patients undergoing HSCT which found crude risk of infection between 29% and 63%. In addition the distribution of infections by aetiology (including the low incidence of fungal infections) reported in these study is roughly similar to the distribution we found [13-16]. This underlines that the incidence and aetiology of infections mainly depend on specific population’s case mix and that, in everyday clinical practice, incidence of infections may be higher than that reported in RCTs designed to assess the effect of interventions in ideal conditions.

Secondly, as reported in other studies [13,17], we found a significant number of viral reactivations suggesting that patients receiving R experience severe cellular-immunity impairment. Cellular-immunity impairment may be unexpected in this patients, as R selectively depleted B-cell (i.e.: humoral-immunity effectors) with virtually no effect on T-cell and macrophage (i.e.: cellular-immunity effectors). This might be explained by two, potentially cooperating, causes. One is that only a limited number of patients underwent R as sole drugs, as all other patients had R in addition to drugs with a well known effects on cellular immunity. A second hypothesis comes from recent studies which suggest that B-cells play a pivotal role in the induction, maintenance and activation of cellular immunity [18]. Clinical data evaluating T-cell response in patients treated with R for autoimmune conditions indicate that the reduction of autoreactive T-cell anticipates and maintains the clinical response in patients with pemphigus [19], idiopathic thrombocytopenic purpura [20], systemic lupus erythematosus [21] and mixed cryoglobulinaemia vasculitis [22]. Moreover the potential effect of B-cells activity upon virus-host interaction has been proven in experimental infection model in mice that showed that the T-Helper 1 specific response against lymphocytic choriomeningitis virus was reduced in a time dependent manner (by 60% on day 8 and by 95% on day 70 post infection) in B cell-depleted mice as compared to naïve mice [23]; in addition another mouse model has shown that T-Helper 1 memory cells recall response to HSV depend on the presence of B cells [24].

Thirdly, the crude temporal analysis found, as expected, that the risk significantly changed over time, being the highest during the earliest phases of therapy and eventually decreasing up to 18 months. In addition we found strong interaction between time and all risk factors significantly associated with infections; namely: HIV status, lymphocytes nadir, GVHD and aggressive lymphoma. In particular HIV + patients and patients with aggressive lymphoma seemed to be at higher risk than others only during the first six month-period while patients with GVHD and those with the lowest lymphocytes nadir seemed to be at greater risk in the latest phases of therapy. The causes behind this observation cannot be explained by our data, but potentially the different patients’ base-line conditions, the different type of protocols they underwent and the B-cell/T-cell interaction described above, may account for this. Additional studies are needed to confirm these observations and provide a better understanding about the relation between patients’ base-line clinical and epidemiological features and the risk of infections.

Finally the frailty model found very strong evidence for clustering with a strong additional risk for subsequent infection(s) after the first infection, both in univariate and in multivariate analyses. This indicated that the study population consisted of a heterogeneous sample of individuals whose different risk for infections is only partially explained by the four risk factors we found significantly associated with the infection.

Several issues may bias our findings. Firstly these data come from a single centre therefore they may not apply the same way to other populations as local epidemiology and/or local policies may vary. Secondly, given the type of data collection, information bias might have affected results in 2 different ways: a) we might have overestimated the number of infections as the consequence of misdiagnosis of colonizations; b) as we conducted a microbiological surveillance we considered only a fraction of all infections which could have actually occurred over the period. However, despite these potential biases, our data are consistent with other published studies.

## Conclusion

In conclusion, although these findings need to be confirmed, we provide preliminary evidence indicating that several patients’ characteristics; i.e.: HIV status, aggressive type of lymphoma, GVHD and low lymphocytes counts at nadir may increase the risk of infection during therapy with R. In addition the effect of these risk factors is significantly modified by time while in therapy. Since Rituximab has been shown to be safe and effective in clinical practice by at least 3 independent systematic reviews [3,4,25], we believe that prospective multicentre cohorts would be the best design to study the different effects of R containing protocols on risk of infections in different groups of patients. The definition of a set of clinically relevant risk factors and a better understating of their relation with time since therapy would greatly improve prevention and clinical management of infections.

## Competing interests

The authors declare that they have no competing interests.

## Authors' contributions

SL designed the study, set up the anonymous database, made the analysis, interpreted results and drafted the manuscript. ACM retrospectively enrolled patients and inputted anonymous database, interpreted results, and reviewed/approved the final text. AGP and GI interpreted results and reviewed/approved the final text. CK approved the study design, interpreted results, reviewed the manuscript and gave final approval to the paper. All authors read and approved the final manuscript.

## Pre-publication history

The pre-publication history for this paper can be accessed here:

http://www.biomedcentral.com/1471-2334/13/317/prepub
